# Bioactivity of Farnesyltransferase Inhibitors Against *Entamoeba histolytica* and *Schistosoma mansoni*

**DOI:** 10.3389/fcimb.2019.00180

**Published:** 2019-05-29

**Authors:** Alexandra Probst, Thi N. Nguyen, Nelly El-Sakkary, Danielle Skinner, Brian M. Suzuki, Frederick S. Buckner, Michael H. Gelb, Conor R. Caffrey, Anjan Debnath

**Affiliations:** ^1^Center for Discovery and Innovation in Parasitic Diseases, Skaggs School of Pharmacy and Pharmaceutical Sciences, University of California, San Diego, La Jolla, CA, United States; ^2^Division of Allergy and Infectious Diseases, Department of Medicine, Center for Emerging and Reemerging Infectious Diseases, University of Washington, Seattle, WA, United States; ^3^Departments of Chemistry and Biochemistry, University of Washington, Seattle, WA, United States

**Keywords:** *Entamoeba histolytica*, *Schistosoma mansoni*, farnesyltransferase, metronidazole, lonafarnib, tipifarnib, statin, chemotherapy

## Abstract

The protozoan parasite *Entamoeba histolytica* can induce amebic colitis and amebic liver abscess. First-line drugs for the treatment of amebiasis are nitroimidazoles, particularly metronidazole. Metronidazole has side effects and potential drug resistance is a concern. Schistosomiasis, a chronic and painful infection, is caused by various species of the *Schistosoma* flatworm. There is only one partially effective drug, praziquantel, a worrisome situation should drug resistance emerge. As many essential metabolic pathways and enzymes are shared between eukaryotic organisms, it is possible to conceive of small molecule interventions that target more than one organism or target, particularly when chemical matter is already available. Farnesyltransferase (FT), the last common enzyme for products derived from the mevalonate pathway, is vital for diverse functions, including cell differentiation and growth. Both *E. histolytica* and *Schistosoma mansoni* genomes encode FT genes. In this study, we phenotypically screened *E. histolytica* and *S. mansoni in vitro* with the established FT inhibitors, lonafarnib and tipifarnib, and with 125 tipifarnib analogs previously screened against both the whole organism and/or the FT of *Trypanosoma brucei* and *Trypanosoma cruzi*. For *E. histolytica*, we also explored whether synergy arises by combining lonafarnib and metronidazole or lonafarnib with statins that modulate protein prenylation. We demonstrate the anti-amebic and anti-schistosomal activities of lonafarnib and tipifarnib, and identify 17 tipifarnib analogs with more than 75% growth inhibition at 50 μM against *E. histolytica*. Apart from five analogs of tipifarnib exhibiting activity against both *E. histolytica* and *S. mansoni*, 10 additional analogs demonstrated anti-schistosomal activity (severe degenerative changes at 10 μM after 24 h). Analysis of the structure-activity relationship available for the *T. brucei* FT suggests that FT may not be the relevant target in *E. histolytica* and *S. mansoni*. For *E. histolytica*, combination of metronidazole and lonafarnib resulted in synergism for growth inhibition. Also, of a number of statins tested, simvastatin exhibited moderate anti-amebic activity which, when combined with lonafarnib, resulted in slight synergism. Even in the absence of a definitive molecular target, identification of potent anti-parasitic tipifarnib analogs encourages further exploration while the synergistic combination of metronidazole and lonafarnib offers a promising treatment strategy for amebiasis.

## Introduction

*Entamoeba histolytica* is a non-flagellated protozoan parasite exclusive to humans that has a simple life cycle comprising an infective cyst stage and an invasive trophozoite form (Petri and Singh, 1999; Stanley, 2003). Infection with *E. histolytica* can lead to three major outcomes: (a) asymptomatic colonization, (b) intestinal amebiasis, most commonly amebic colitis, and (c) extra-intestinal amebiasis with liver abscess being the most common complication (Petri and Singh, 1999). Amebiasis causes up to 110 thousand deaths annually and is estimated to be the second most common cause of parasite infection-related mortality worldwide (Petri and Singh, 1999; Lozano et al., 2012; Watanabe and Petri, 2015). Each year 40 to 50 million cases of amebic colitis and liver abscess are reported with high prevalences in Central and South America, Africa, and Asia (Petri and Singh, 1999).

Amebic infection is initiated by ingestion of *E. histolytica* cysts in fecally contaminated food or water. These cysts excyst in the intestine to form trophozoites, which degrade the mucous layer via cysteine protease activities, destroy and ingest epithelial cells via trogocytosis, and invade the lamina propria, which leads to colitis and liver abscesses in the case of invasion of the blood vessels (Petri, 2002; Stauffer and Ravdin, 2003; Watanabe and Petri, 2015).

First-line drugs for the treatment of invasive amebiasis are the nitroimidazoles, in particular metronidazole, which is given orally to adults in three doses of 750 mg (total 2,250 mg/day) per day for 7–10 days (Haque et al., 2003). Nitroimidazole compounds carry a nitro group on the 5-position of the imidazole ring. As prodrugs, that must be activated by reductases of the parasite. After entering the trophozoite, reduced ferredoxin donates electrons to the nitro group of the prodrug, which is then reduced to toxic radicals. Covalent binding to DNA macromolecules results in DNA damage and killing of the parasites (Muller, 1983; Edwards, 1993). Nitroreductases and thioredoxin reductase are also known to reduce nitroimidazole drugs in *Entamoeba* (Leitsch et al., 2007).

Potential resistance of *E. histolytica* to metronidazole remains a major concern (Samarawickrema et al., 1997; Wassmann et al., 1999) and in the absence of a back-up drug, it is important to search for alternative antimicrobials against *E. histolytica*.

Schistosomiasis is caused by various species of the *Schistosoma* flatworm that reside in the venous system. Infection is found in populations living close to freshwater bodies that harbor the appropriate vector snail. With as many as 200 million people infected (Hotez, 2018) and possibly over 700 million at risk (King, 2010), infections can be chronic and painful as a consequence of progressive tissue and organ damage due to the parasite's eggs. The disease impacts school attendance and performance, the ability to work, and, consequently, it has been considered a direct contributor to poverty (Hotez et al., 2008; Utzinger et al., 2011). Treatment and control of schistosomiasis relies on just one drug, praziquantel. Though safe and reasonably effective, the drug is rarely curative and is less effective against immature parasites (Caffrey, 2007, 2015). The possibility of resistance, particularly as dissemination of the drug is increasing (http://unitingtocombatntds.org/wp-content/themes/tetloose/app/staticPages/fifthReport/files/fifth_progress_report_english.pdf, 2014) is a constant concern, and alternative drugs would be welcome.

The mevalonate metabolic pathway is vital for diverse functions in parasitic protozoa and helminths such as sterol synthesis and cell growth (Li et al., 2013; Rojo-Arreola et al., 2014; Millerioux et al., 2018). 3-hydroxy-3-methyl-glutaryl-coenzyme A reductase (HMGR) is the rate-limiting enzyme in the pathway which catalyzes the conversion of HMG-CoA into mevalonate (Edwards and Ericsson, 1999). HMGR inhibitors, also known as statins, prevent the conversion of HMG-CoA to l-mevalonate resulting in the inhibition of the downstream sterol biosynthesis (Gazzerro et al., 2012). Mevalonate is also a precursor of isoprenoid groups, in addition to more than a dozen classes of end products (Goldstein and Brown, 1990). The last common substrate for the synthesis of the end products of the mevalonate pathway is farnesyl pyrophosphate (FPP, also referred to as farnesyl diphosphate FDP). It is the natural substrate of farnesyl transferase (FT), which catalyzes the transfer of a farnesyl moiety from FPP to proteins. Farnesylated proteins include Ras and Ras related GTP-binding proteins, nuclear lamins, centromere-associated proteins, tyrosine phosphatases, and co-chaperones (Zhang and Casey, 1996). FT catalyzes the prenylation of proteins through a thioether linkage and the prenylation may be concluded by the palmitoylation of cysteine residues for some proteins. Due to the lipids involved in the mechanism and their hydrophobicity, prenylation leads to membrane interactions by the proteins and plays an important role in the signal transduction pathway for cell differentiation (Zhang and Casey, 1996). A previous characterization of *E. histolytica* FT showed that the amebic FT did not utilize a majority of Ras and Rap as substrates, but only one Ras protein, Ras4, was farnesylated (Kumagai et al., 2004). *Schistosoma mansoni* Ras was also found to be farnesylated and inhibition of farnesylation in *S. mansoni* extract was achieved using an FT inhibitor (FTI) (Osman et al., 1999). The deduced amino acid sequences of the β-subunit of both *E. histolytica* and *S. mansoni* FT are 36 and 43% identical to the β-subunit of human FT. There is evidence that targeting the farnesyltransferase enzyme in protozoan parasites leads to inhibition of protein prenylation and severely impairing growth, including *Plasmodium falciparum* (Ibrahim et al., 2001; Buckner et al., 2002; Chakrabarti et al., 2002; Carrico et al., 2004; Esteva et al., 2005). Here, we demonstrate the anti-parasitic activities of known FTIs lonafarnib and tipifarnib against *E. histolytica*, and *S. mansoni* somules (post-infective larvae) and adults. In addition, we identified tipifarnib analogs with anti-amebic and anti-schistosomal activities. The combination of lonafarnib with the currently used anti-amebic drug, metronidazole, generated a synergistic growth inhibition of *E. histolytica*.

## Materials and Methods

### Chemicals and Reagents

**Figure 1:**
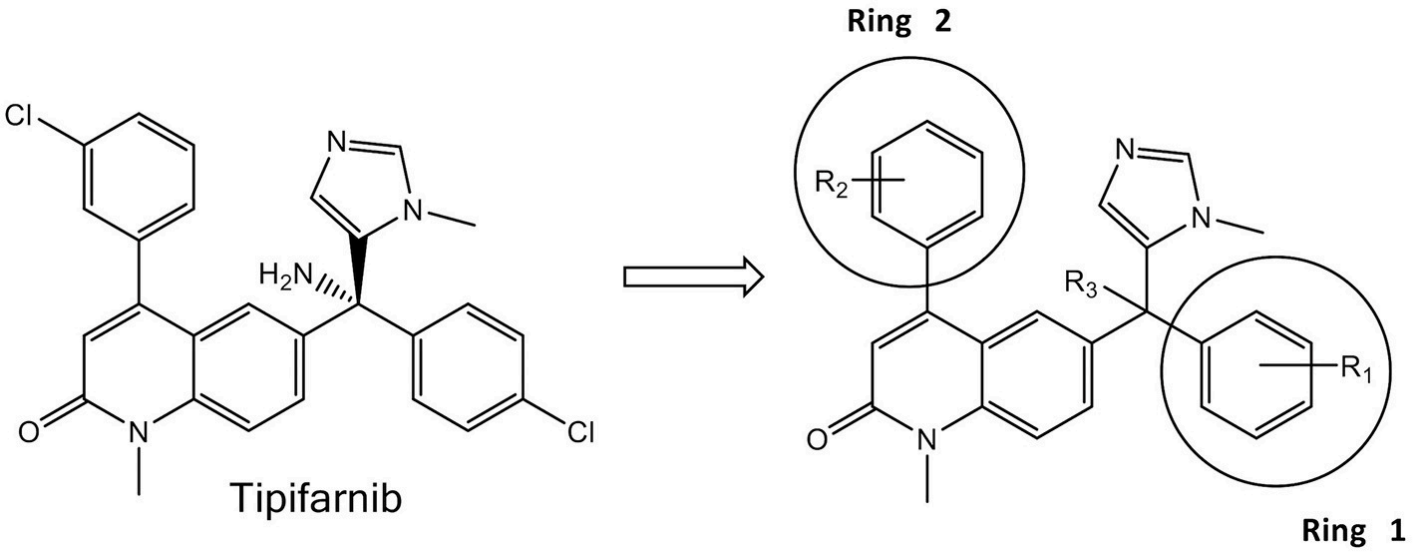
Tipifarnib **(Left)** and the generic structure of 125 variants **(Right)** tested against *E. histolytica* and *S. mansoni*.

Assay plates (format includes: 96- and 24-well; flat and U-bottomed; and transparent and white) were purchased from VWR International (Radnor, PA). The CellTiter-Glo luminescent cell viability assay was acquired from Promega (Madison, WI); DMSO, metronidazole, lonafarnib and simvastatin were purchased from Sigma-Aldrich (St. Louis, MO). The tipifarnib analogs (Figure 1) were previously synthesized as part of a program to develop FTIs active against *Trypanosoma brucei* and *Trypanosoma cruzi*, the causative agents of Human African Trypanosomiasis and Chagas disease, respectively (Kraus et al., 2009, 2010). Compounds were dissolved in DMSO at a concentration of 10 mM and stored at −20°C.

### Maintenance of *Entamoeba histolytica*

*E. histolytica* trophozoites (strain HM1:IMSS) were maintained axenically in TYI-S-33, supplemented with penicillin (100 U/mL), streptomycin (100 μg/mL) and 10% heat inactivated adult bovine serum as previously described (Diamond et al., 1978). The cells were maintained in the logarithmic phase of growth by routine passage every 2 days and the logarithmic phase of growth was determined by counting the cells using a hemocytometer.

### Maintenance of *Schistosoma mansoni*

The NMRI isolate of *S. mansoni* was maintained by passage through *Biomphalaria glabrata* snails and 3–5 week-old, female Golden Syrian hamsters (Charles River, San Diego, CA) as intermediate and definite hosts, respectively (Abdulla et al., 2009; Long et al., 2016). A dose of 600 infective larvae (cercariae) was used to infect hamsters. The acquisition, preparation and *in vitro* maintenance of *S. mansoni* post-infective larvae (schistosomula or somules) and adults have been described (Abdulla et al., 2009; Štefanić et al., 2010). Vertebrate maintenance and handling at the University of California San Diego Animal Care Facility were in accordance with protocols approved by the university's Institutional Animal Care and Use Committee (IACUC).

### First-Pass Screening of the FTIs and Statins for Activity Against *Entamoeba histolytica*

A first-pass cell viability assay was performed with compounds at a concentration of 50 μM against *E. histolytica* (Debnath et al., 2014). Briefly, 0.5 μL of 10 mM (FTIs) or 20 mM (statins) stock compounds were plated into white 96-well flat-bottom plates in duplicate and 5,000 trophozoites in 99.5 μL TYI-S-33 medium were added to the 96-well plates. As a positive control, 50 μM of metronidazole were plated; negative controls contained 0.5% of DMSO. Cell culture plates were incubated at 37°C for 48 h in the GasPak EZ gas-generating anaerobe pouch system (VWR). After incubation, 50 μL of CellTiter-Glo^®^ were added. The luminescent signal, resulting from the lysis of the cells was measured by an EnVision luminometer, and can be converted into the percentage of inhibition of the cell growth relative to maximum and minimum reference signal controls by using the following equation (Debnath et al., 2012):

$$\begin{array}{l} \%\;Inh.\;=\left(1-\left(\frac{\left(luminescent\;output\;\left(RLU\right)of\;compound\;x-mean\;of\;luminescent\;output\;\left(RLU\right)of\;positive\;control\right)}{\left(mean\;of\;luminescent\;output\;\left(RLU\right)of\;negative\;control\;-\;mean\;of\;luminescent\;output\;\left(RLU\right)of\;positive\;control\right)}\right)\right)*100\; \end{array}$$

### Dose-Response Assays of the FTIs Lonafarnib and Tipifarnib, Simvastatin as Well as Metronidazole Against *Entamoeba histolytica*

Dose-response assays were implemented as confirmatory screens of FTIs, simvastatin and metronidazole against *E. histolytica* trophozoites. An 8-point EC_50_ determination (half maximal effective concentration) was performed as follows: 0.5 μL of 10 mM (FTIs) or 20 mM (simvastatin) stock compounds was plated in triplicate into white 96-well flat-bottom plates to obtain a starting concentrations of 50 or 100 μM. Compounds were then serially diluted in triplicate to obtain a concentration range of 50 to 0.39 μM for FTIs and 100 to 0.78 μM for simvastatin. The addition of the cells, the incubation and the reading were performed as described in the first-pass assay. Graph, EC_50_ calculations and standard error (fitting method: least square (ordinary) fit) were obtained by using GraphPad Prism (San Diego, CA) (Debnath et al., 2012).

### Mammalian Cytotoxicity Assay

Tipifarnib was screened for cytotoxicity against the fibroblast 3T3 cell line. Cells were grown in the presence of tipifarnib for 48 h before growth was quantified using Alamar Blue (Alamar Biosciences Inc., Sacramento, CA) (Kraus et al., 2009). Tipifarnib was tested at final concentrations of 100, 50, 25, 12.5, 6.25, 3.13, 1.56, and 0.78 μM.

### *Entamoeba histolytica* Invasion Assay

This was performed as described previously (Emmanuel et al., 2015). Briefly, 75,000 *E. histolytica* trophozoites were pre-incubated for 3 h with 0.5% DMSO, 14 and 25 μM of tipifarnib and after 3 h, cells were re-suspended in serum-free TYI medium and loaded in the upper chamber of a Corning BioCoat Matrigel Invasion Chamber (Corning). The lower chamber contained TYI medium supplemented with 10% adult bovine serum (Sigma-Aldrich). The Matrigel Invasion Chamber was incubated at 37°C for 48 h in a GasPak EZ gas-generating anaerobe pouch system. At the end of incubation, images of trophozoites in the lower chamber were captured using a 10 × objective lens fitted in a Zeiss Axiovert A1 inverted microscope. Trophozoites that had migrated into the lower chamber were also counted using a hemocytometer. The data were obtained from three independent experiments each performed in triplicate and the percentage invasion of trophozoites was calculated and plotted by using GraphPad Prism.

### Combination Assays

Because the activity of tested FTIs and simvastatin against *E. histolytica* was moderate when administered alone, we tested if a combination of lonafarnib with metronidazole or an HMG-CoA reductase inhibitor (simvastatin) would have a better activity against *E. histolytica in vitro* than a single compound. Drugs were combined at constant ratios 1:1, 1:2, 1:4, 1:8, 2:1, 4:1, 8:1, 16:1 [μM lonafarnib/μM metronidazole and μM lonafarnib/μM simvastatin]. Growth inhibition was determined with the CellTiter-Glo® luminescent cell viability assay, as described previously (Debnath et al., 2012). Quantitative drug interaction (synergy) was determined by using the software CompuSyn (Chou and Talalay, 1984; Chou, 2006).

### Phenotypic Evaluation of the Effect of Combination of Lonafarnib and Metronidazole on *Entamoeba histolytica* Trophozoites

The assay was performed using transparent 96-well flat-bottom plates. DMSO was used as a negative control at a final concentration of 0.5%. Metronidazole was plated at 50 μM as a positive control. Lonafarnib and metronidazole were combined at ratios of 4:1, 2:1, 1:1, 1:2, and 1:4. Parasites were plated and incubated for 48 h as described in the first pass and dose response assay for *E. histolytica* (Debnath et al., 2014). Images were captured after 24 and 48 h post-incubation using a Zeiss Axiovert A1 inverted microscope (10 ×, 20 ×, 40 × and 63 × objective) and a Zeiss AxioCam 503 mono digital camera controlled by the Zen 2 lite software (Version 2.0.0.0).

### Phenotypic Screening of FTIs With Different Developmental Stages of *Schistosoma mansoni*

Screens were performed using post-infective larvae (somules) and adult parasites, as described (Abdulla et al., 2009; Rojo-Arreola et al., 2014; Long et al., 2016) using transparent U-shaped 96-well plates and flat-bottom 24-well plates, respectively. Given the relatively large number of compounds to screen, the entire collection was screened against somules to identify the most active compounds for subsequent screening against adult parasites, as conducted previously (Abdulla et al., 2009). Somules are harvestable in their thousands from vectors snails whereas adults are only recoverable from infected hamsters in limited numbers (~10–20% return on the infecting 600 cercarial dose used).

Somules (40 units/well) were prepared in 100 μL Basch medium (Basch, 1981) supplemented with 5% FBS, 100 U/mL penicillin and 100 μg/mL streptomycin. Compounds were then added at 2x the final concentration in 100 μL of the same medium to yield final concentrations of 5 or 10 μM compound and 0.5% DMSO. Parasites were incubated at 37°C in a 5% CO_2_ environment and phenotypic changes recorded at 24 and 48 h. Adult 42-day-old *S. mansoni* (5 males and including approximately 2 pairs) was maintained in 2 ml of the same Basch medium under the same conditions in the presence of 10 μM compound and 0.1% DMSO. Phenotypic changes were recorded at 1, 5, 24, and 48 h.

Phenotypic changes are recorded as described (Abdulla et al., 2009; Rojo-Arreola et al., 2014; Glaser et al., 2015; Weeks et al., 2018). Briefly, we employ simple descriptors that describe the effects of compounds on the parasites (changes in shape, motility, and density). To allow for comparisons of compound activity, each descriptor is awarded a “severity score” of 1 and these are added up to a maximum score of 4. Evidence of degeneracy or death is awarded the maximum score of 4, and, for adults specifically, non-adherence by the oral or ventral suckers to the well surface (a score of 1) is taken into account as is damage to the outer surface (tegument; a score of 4) on the understanding that such damage is lethal *in vivo* to the parasite (Andrews et al., 1983). Images were captured via a Zeiss AxioCam 105 color digital camera that was attached to a Zeiss Axiovert A1 inverted microscope and controlled by ZEN 2 lite software (Version 2.0.0.0).

## Results

### First-Pass Screening of FTIs (Lonafarnib, Tipifarnib, 125 Analogs of Tipifarnib) and Statins Against *Entamoeba histolytica*

Table 1 lists the most active statins and FTIs that were obtained from the first pass screening against *E. histolytica* trophozoites (>75% growth inhibition). From the five statins tested (mevastatin, atorvastatin, fluvastatin, pitavastatin, simvastatin), only simvastatin exhibited 94% growth inhibition at 100 μM. Lonafarnib, tipifarnib and 17 tipifarnib analogs (Table 1) demonstrated >75% of inhibition at 50 μM after an incubation period of 48 h. Both lonafarnib and tipifarnib demonstrated almost 100% inhibitory activity and one tipifarnib analog, HB-24 showed 98.9% inhibition at 50 μM. The results of the first-pass screening against *E. histolytica* trophozoites for all 125 tipifarnib analogs are shown in Supplementary Table 1. Activities were stratified into four groups depending on the percentage growth inhibition of *E. histolytica* trophozoites. Compounds showing 75–100% growth inhibition belonged to group A, group B showed 50–74% inhibition, group C had 25–49% growth inhibition and group D exhibited 0–24% growth inhibition. In referencing to the generic structure shown in Figure 1 (right), most of the active compounds contain R_3_ = NH_2_ (11 of 17). For the analogs, ring 1 was either left the same as tipifarnib (4-Cl) or modified to 4-CH_3_, 4-CF_3_, 3-Cl, or several other variants. There were no apparent trends associating specific ring 1 substituents with more potent *E. histolytica* activity. With respect to ring 2, compounds with the 3-Cl-phenyl (as occurs with tipifarnib) were found in groups 1, 2, 3, and 4.

**Table 1:** Activity of statins at 100 μM and tipifarnib analogs at 50 μM against *E. histolytica*.

**Compound name**	**Growth inhibition (%)**
Simvastatin	94
Tipifarnib	99
Lonafarnib	100
CHN-12	77.1
CHN-13 CHN-14	86.6 77.1
HB-15	78.6
HB-21 HB-24 HB-27	90.2 98.9 95.5
HB-30	86.3
JK-02	75.1
JK-12	91.5
JK-13	83.2
JK-17 JK-19	92.6 81.9
JK-21	77.0
JK-31 PN-103	84 77.0
PN-133	78.0

### *Entamoeba histolytica* Concentration Response Assay

**Figure 2:**
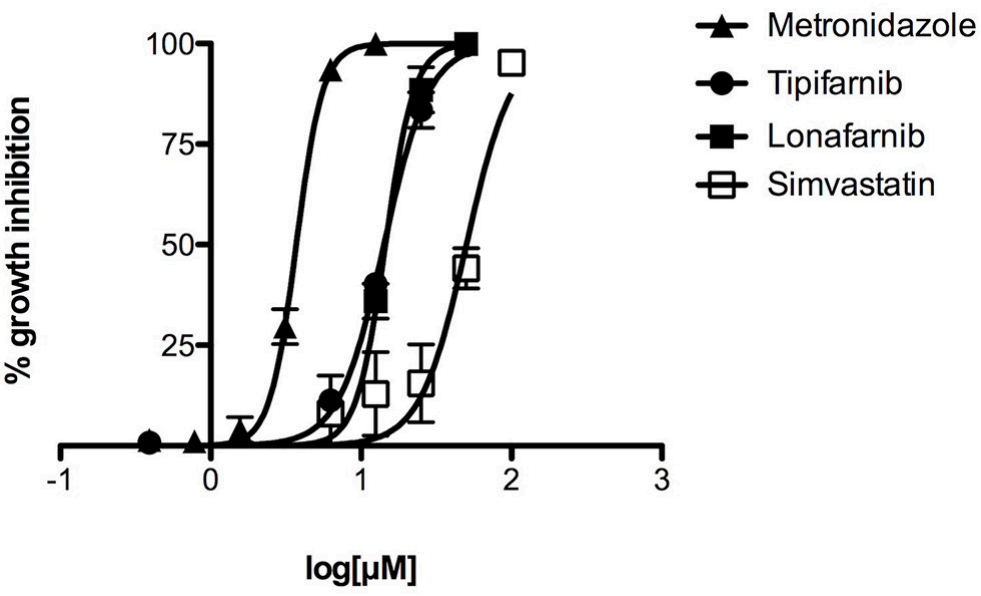
Concentration-dependent inhibition of growth of *E. histolytica* by tipifarnib, lonafarnib, and simvastatin, as compared to metronidazole. Different concentrations of compounds were tested in triplicate for activity against *E. histolytica* trophozoites. The data points represent mean percentage growth inhibition and standard error of mean (SEM) of different concentrations of metronidazole, tipifarnib, lonafarnib and simvastatin. EC_50_ curves were generated from mean values of percentage growth inhibition ± SEM of metronidazole, tipifarnib, lonafarnib, and simvastatin against *E. histolytica*.

As simvastatin and FTIs tipifarnib and lonafarnib were among the most active compounds in the primary screen, they were selected for further testing. Some of the active tipifarnib analogs containing NH2 at R_3_ were also assessed for EC_50_. The EC_50_ of simvastatin was 50 μM, whereas both tipifarnib and lonafarnib generated EC_50_ values of about 14 μM. The EC_50_ value for tipifarnib on a mammalian fibroblast cell line 3T3 was 35.1 μM. This provided a selectivity index of about 2.5. The most active tipifarnib analog in the primary screen, HB-24, generated an EC_50_ value similar to those of lonafarnib or tipifarnib (Table 2). The dose response and EC_50_ data for simvastatin, tipifarnib, lonafarnib, and metronidazole are displayed in Figure 2 and Table 2, respectively.

**Table 2:** EC_50_ values of simvastatin, FTIs, tipifarnib analogs, and metronidazole against *E. histolytica*.

**Compound name**	**EC_**50**_ ± SE (μM)^**a**^**
Simvastatin	50.2 ± 0.05
Tipifarnib	14.2 ± 0.02
Lonafarnib	14.5 ± 0.05
CHN-13	27.6 ± 0.03
HB-21	26.4 ± 0.03
HB-24	17.5 ± 0.03
JK-17	20.2 ± 0.03
JK-31	26.2 ± 0.01
Metronidazole	3.7 ± 0.01

a *EC_50_ minimum n = 3*.

### *Entamoeba histolytica* Invasion Assay

**Figure 3:**
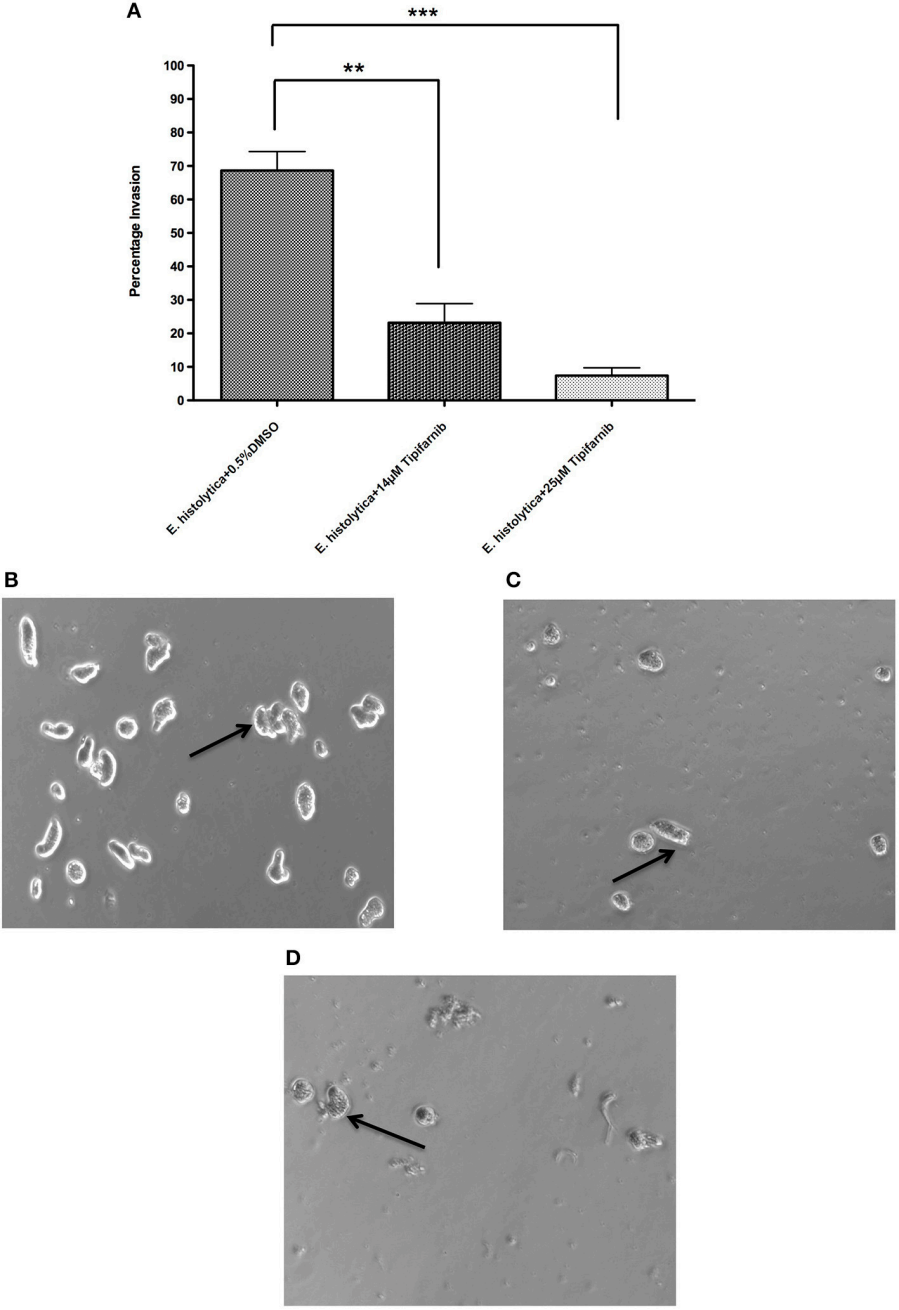
Effect of tipifarnib on invasion of matrigel by *E. histolytica*. *E. histolytica* trophozoites were incubated for 3 h with 0.5% DMSO, 14 μM of tipifarnib and 25 μM of tipifarnib and at the end of incubation trophozoites were allowed to invade the matrigel for 48 h. After 48 h, the migrated cells in the lower chamber were counted and percentage invasion of trophozoites was calculated and plotted **(A)**. Images of trophozoites at 48 h were captured using a Zeiss Axiovert A1 inverted microscope and a Zeiss AxioCam 503 mono digital camera controlled by Zen 2 lite software. Trophozoites were treated for 3 h with 0.5% DMSO **(B)**, 14 μM tipifarnib **(C)**, and 25 μM tipifarnib **(D)** and migrated trophozoites in lower chambers were imaged at 48 h. Trophozoites were photographed at 10 × magnification. Arrow indicates *E. histolytica* trophozoites. **P < 0.01 and ***P < 0.001 by Student's *t*-test compared to 0.5% DMSO-treated *E. histolytica* trophozoites.

To test whether tipifarnib influences invasiveness of *E. histolytica*, we used a transwell matrigel invasion assay. The data show that a 3 h pre-incubation of trophozoites with tipifarnib at its EC_50_ concentration (14 μM) or at 25 μM decreased the invasion of trophozoites through the matrigel after 48 h compared to 0.5% DMSO-treated trophozoites (Figure 3). Specifically, whereas 68.7 ± 5.7% of the DMSO-treated trophozoites penetrated the matrigel and were recovered in the lower chamber, just 23.2 ± 5.7% (*p* = 0.005) and 7.3 ± 2.3% of the cells (*p* = 0.0005) that had been exposed to 14 and 25 μM tipifarnib were recovered, respectively.

### Synergy Assay of Lonafarnib and Metronidazole Against *Entamoeba histolytica*

To measure the effect of combining lonafarnib and metronidazole, drugs were combined at constant micromolar ratios and the inhibition of *E. histolytica* trophozoites growth was determined using the CellTiter-Glo® luminescent cell viability assay. Synergism was observed for all combinations of lonafarnib and metronidazole at 50, 75, 90, and 95% of growth inhibition, respectively. Synergism at high effect level is much more relevant than at low effect level, therefore a 95% growth inhibition of *E. histolytica* trophozoites upon drug administration was chosen to be presented in Table 3. All tested combinations of lonafarnib and metronidazole showed synergism, given the combination index (CI) values being <1. According to Chou (Chou, 2006), CI values ranging from 0.85 to 0.9 indicate slight synergism, values in the range of 0.7 to 0.85 and 0.3 to 0.7 indicate moderate synergism and synergism, respectively. Combining lonafarnib and metronidazole led to a reduction of each dose (Dose Reduction Index or DRI) at given effect level compared with the doses of each drug alone.

**Table 3:** Summary of synergism assay with lonafarnib and metronidazole, shown for 95% growth inhibition of *E. histolytica* trophozoites.

**Drug combination ratio**	**% growth inhibition**	**CI**	**DRI**		**Dose required (μM)**	
**Lonafarnib:metronidazole**			**Lonafarnib**	**Metronidazole**	**Lonafarnib**	**Metronidazole**
1:1	95	0.8 ± 0.04	10.1 ± 0.91	1.4 ± 0.03	9.4 ± 0.79	9.4 ± 0.79
1:2	95	0.7 ± 0.02	21.1 ± 2.6	1.4 ± 0.02	4.6 ± 0.29	9.1 ± 0.57
1:4	95	0.6 ± 0.12	53.8 ± 20.94	1.7 ± 0.37	1.9 ± 0.27	7.6 ± 1.08
1:8	95	0.6 ± 0.04	97.5 ± 17.17	1.6 ± 0.1	0.7 ± 0.49	5.6 ± 3.91
2:1	95	0.8 ± 0.05	8.2 ± 4.71	1.5 ± 0.05	17.6 ± 1.23	8.8 ± 0.62
4:1	95	0.7 ± 0.13	5.7 ± 3.62	2 ± 0.1	25.9 ± 0.65	6.5 ± 0.17
8:1	95	0.5 ± 0.14	5 ± 3.08	3.5 ± 0.23	29.7 ± 0.02	3.71 ± 0.01
16:1	95	0.6 ± 0.29	3.5 ± 2.43	4.8 ± 1.01	44.7 ± 7.5	2.8 ± 0.47
	95				95.4 ± 19.7	13 ± 0.9

*Mass-action law based computer software “CompuSyn” was used for automated data analysis. Experiments were carried out in triplicate. CI stands for combination index, DRI for dose reduction index*.

### Phenotypic Evaluation of the Effect of the Synergy Experiment With Lonafarnib and Metronidazole on *Entamoeba histolytica* Trophozoites

**Figure 4:**
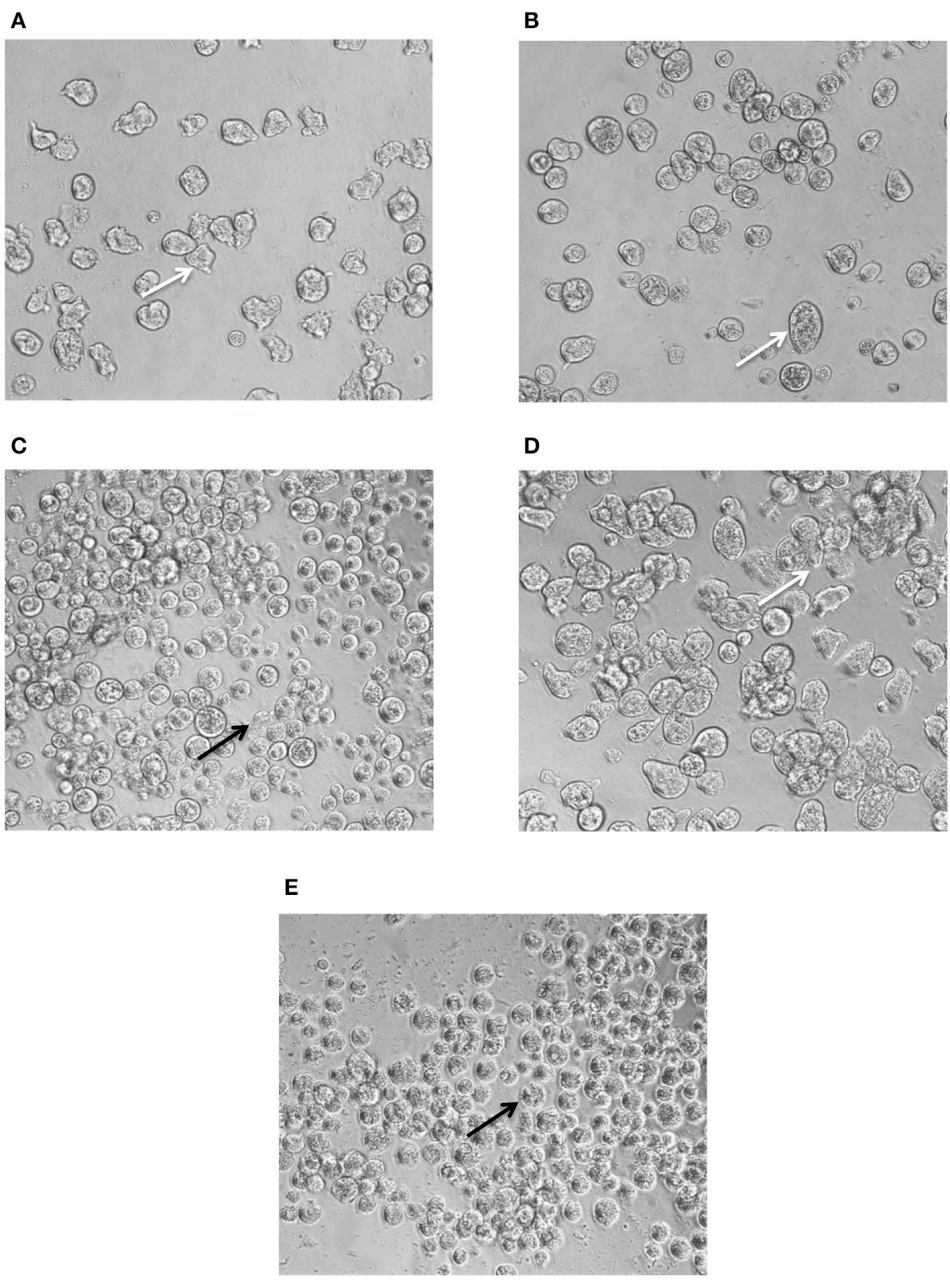
Phenotypic changes in *E. histolytica* upon administration of FTI lonafarnib in combination with metronidazole. *E. histolytica* trophozoites were photographed 24 h after treatment with lonafarnib and metronidazole using a Zeiss Axiovert A1 inverted microscope and a Zeiss AxioCam 503 mono digital camera controlled by Zen 2 lite software. The trophozoites were treated with 7.4 μM of lonafarnib alone **(A)**, 1.9 μM of metronidazole alone **(B)**, a combination of 7.4 μM of lonafarnib and 1.9 μM of metronidazole **(C)**, 0.5% DMSO **(D)** and 50 μM of metronidazole **(E)**. White arrow indicates normal morphology of trophozoites and black arrow indicates rounded, lysed trophozoites. Trophozoites were photographed at 20 × magnification.

Trophozoites treated with 7.4 μM of lonafarnib (Figure 4A) showed a similar morphology and movement to 0.5% DMSO-treated control trophozoites (Figure 4D) after 24 h of incubation. This concentration of lonafarnib is almost half of the concentration needed for a 50% cell growth inhibition (EC_50_ = 14.5 μM). Treatment with metronidazole alone (1.9 μM; Figure 4B) evoked the same phenotypic response as reported for lonafarnib. When lonafarnib and metronidazole were administered in a combination ratio of 4:1 (7.4 μM lonafarnib: 1.9 μM metronidazole) (Figure 4C), lysis of the cells was induced and completed by 24 h post-incubation. The cell death observed at 24 h in lonafarnib-metronidazole combination experiment was comparable to the death induced by 50 μM of metronidazole alone (Figure 4E).

### Effect of Combination of Lonafarnib and Simvastatin Against *Entamoeba histolytica*

The inhibitory effects of lonafarnib and simvastatin were estimated by ATP-bioluminescence assay at fixed concentration ratios, and their dose-effect relationships were assessed by Chou-Talalay combination index (CI) method using CompuSyn software. Only two ratios of lonafarnib and simvastatin (1:1 and 2:1) showed slight synergism to additivity with CI ≤ 1 for 50, 75, 90, and 95% of growth inhibition. The combination of lonafarnib and simvastatin at 1:1 and 2:1 achieved 95% growth inhibition with 1.2- to 1.4-fold dose reduction for lonafarnib and 4- to 6.4-fold dose reduction for simvastatin (Table 4).

**Table 4:** Summary of combination assay with lonafarnib and simvastatin, shown for 95% growth inhibition of *E. histolytica* trophozoites.

**Drug combination ratio**	**% growth inhibition**	**CI**	**DRI**		**Dose required (μM)**	
**Lonafarnib:simvastatin**			**Lonafarnib**	**Simvastatin**	**Lonafarnib**	**Simvastatin**
1:1	95	1 ± 0.09	1.4 ± 0.37	4 ± 1.68	15.9 ± 3.05	48 ± 24.82
2:1	95	0.9 ± 0.12	1.2 ± 0.4	6.4 ± 2.05	15.9 ± 3.05	48 ± 24.82

*Mass-action law based computer software “CompuSyn” was used for automated data analysis. Experiments were carried out in triplicate. CI, combination index; DRI, dose reduction index*.

### Phenotypic Screening of FTIs Against *Schistosoma mansoni*

Because the anti-schistosomal activity of statins, including mevastatin, atorvastatin, fluvastatin and simvastatin, has already been demonstrated against *S. mansoni* somules and adults (Rojo-Arreola et al., 2014; Asarnow et al., 2015), the phenotypic screening in this study focused on the FTIs.

**Figure 5:**
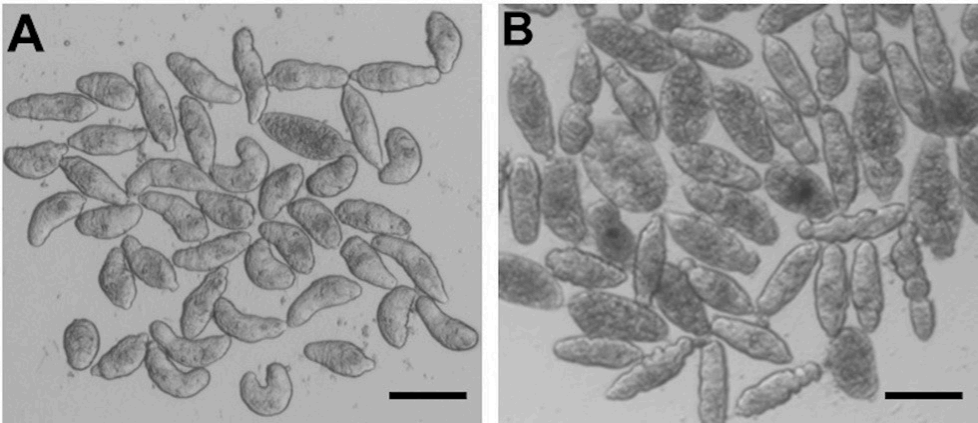
*S. mansoni* somules incubated for 48 h as described in the text. **(A)** DMSO control; **(B)** in the presence of 10 μM tipifarnib—note the rounding and varying degrees of degeneracy of the parasites. Scale bar = 200 μm.

For somules, phenotypic changes in the presence of the FTIs were assessed visually after 24 and 48 h at 5 and 10 μM (Figure 5 as a representative example), and the descriptors recorded were converted to severity scores to allow for comparison of compound activities. Based on the combination of time- and dose-dependency of activity, the compounds could be ranked into four groups (Groups A-D; Supplementary Table 2). Group A comprised the 17 most active compounds that generated severity scores of 3 or 4 at either 5 or 10 μM after 48 h (Table 7). These included lonafarnib and tipifarnib. Group B comprised 47 compounds that were maximally active (score of 4) at both 24 and 48 h at 10 μM, but elicited little or no activity at 5 μM. The 34 Group C compounds were those for which severity scores of 1–3 were recorded at 10 μM after 24 h and, finally, the 28 Group D compounds were those that registered zero scores at 10 μM after 24 h. Throughout, the most common phenotypic responses recorded were loss of translucency (darkening) and degeneracy sometimes leading to death.

The most active 17 Group A compounds were subsequently screened at 10 μM against 42-day-old adult *S. mansoni* and phenotypes recorded at 1, 5, 24, and 48 h. All were active to varying degrees (Table 7, Supplementary Table 2, Supplementary Videos 1, 2). Thus, the most common phenotypic responses of the parasites noted at 1 and 5 h was an uncoordinated motion coupled with an inability of the parasite to adhere via its oral or ventral suckers to the floor of the well (severity score of 2). By 24 and 48 h, for the 12 most active compounds, these responses had progressed to degeneracy, worm shrinkage, decreased motility and blebbing of the surface tegument (severity scores of 3 or 4). Among the Group A compounds, and apart from lonafarnib and tipifarnib, only CHN-13, JK-02, JK-17, and JK-19 overlapped with those compounds that were most active against *E. histolytica* (Table 1).

Analysis of the physicochemical properties of all 125 compounds indicated that there was a statistically significant trend for compounds with higher cLogP values being associated with better *E. histolytica* and *S. mansoni* activity (Tables 5, 6).

**Table 5:** Average cLogP values for FTIs according to group^*^ in *E. histolytica*.

**Group**	**A**	**B**	**C**	**D**
No. of compounds	17	25	40	40
cLogp (mean)	5.14	4.9	5.06	4.26
SE	0.19	0.18	0.15	0.20

* *Groups A, B, and C each have cLogP values that are significantly higher than group D (P < 0.05, ANOVA)*.

**Table 6:** Average cLogP values for FTIs according to group^*^ in *S. mansoni*.

**Group**	**A**	**B**	**C**	**D**
No. of compounds	17	47	34	28
cLogp (mean)	5.32	5.28	4.89	3.66
SE	0.22	0.10	0.17	0.20

* *Groups A, B, and C each have cLogP values that are significantly higher than group D (P < 0.001, ANOVA)*.

## Discussion

We report the anti-parasitic activities of FTIs against two phylogenetically distinct parasites, the protozoan, *E. histolytica*, and the metazoan flatworm, *S. mansoni*. FT has gained much attention as a target for pharmaceutical development and a number of FTIs have been tested as anti-cancer agents (Bagchi et al., 2018). Both lonafarnib and tipifarnib are potent inhibitors of mammalian FT and have been extensively tested in clinical trials for malignancies (Martinelli et al., 2008; Moorthy et al., 2013). Lonafarnib has also undergone clinical trials in children with progeria (Gordon et al., 2016). Thus, in an observational cohort study in patients with progeria, lonafarnib treatment led to a significantly reduced mortality rate after 2.2 years of follow-up (Gordon et al., 2018).

The role of protein farnesylation has been previously documented in *E. histolytica* (Kumagai et al., 2004). Specifically, the *E. histolytica* Ras4 (a small GTPase mediating cell proliferation/differentiation) was identified as a unique farnesyl acceptor for the *E. histolytica* FT (Kumagai et al., 2004). Molecular characterization of protein farnesyltransferase in *E. histolytica* demonstrated that α and β subunits are well-conserved signature domains shared by other organisms (Kumagai et al., 2004).

The studies reported herein demonstrate that two FTIs, lonafarnib and tipifarnib, were active against *E. histolytica* in culture with EC_50_ values of ~14 μM. Because the compounds are less potent than the current standard metronidazole therapy, we screened a library of 125 tipifarnib analogs against *E. histolytica* in culture to identify compounds with greater potency and a structure-activity relationship (SAR). This library was originally assembled to target the protozoan parasite, *Trypanosoma cruzi*, and comprised compounds with known FTI activity as well as compounds with structural changes that abrogated that activity (Kraus et al., 2009, 2010). The changes were intended to reduce activity on the mammalian FT in order to avoid potential side-effects, while retaining anti-parasitic activity that was mediated through inhibition of the *T. cruzi* CYP51 enzyme (essential for ergosterol metabolism) (Kraus et al., 2009). The methylimidazole attached to the central sp3 carbon (Figure 1) is integral to the activity against both FT and sterol 14-alpha-demethylase (CYP51), and is retained in all of the analogs. However, changes to other substituents at positions R1–R3 (Figure 1, right) resulted in decreased FTI activity compared to tipifarnib (mammalian FT IC_50_ value of 0.7 nM). Specifically, introduction of a 2-methyl group on Ring 2 or replacing the tipifarnib Ring 2 with a naphthyl group yielded an IC_50_ value of 294 or 485 nM, respectively, against mammalian FT (Kraus et al., 2009). Combining the Ring 2 changes with a change of the NH_2_ to OMe at the R3 position essentially abrogated FTI activity (mammalian FT IC_50_ of >5,000 nM).

Previously reported whole-organism studies *in vitro* with tipifarnib demonstrated moderate bioactivity (EC_50_ = 6 μM) against the protozoan parasite, *Trypanosoma brucei* (Buckner et al., 2005), but much more potent activity against *Trypanosoma cruzi* (EC_50_ = 0.004 μM) (Kraus et al., 2010). Against the *Trypanosoma brucei* FT recombinantly expressed in *E. coli* (Buckner et al., 2000), tipifarnib analogs with R_3_ = NH_2_ (e.g., JK-02, JK-12, JK-13, JK-17, and JK-19), i.e., those that were active against *E. histolytica*, possess weak to negligible activity (data not published). Further, we previously demonstrated that 11 of the 17 most active tipifarnib analogs against *E. histolytica* and which contained the same NH_2_ R_3_ group (Supplementary Table 1) exert their activity against *T. cruzi* via inhibition of CYP51 (Kraus et al., 2009), yet, a CYP51 ortholog is not found in the *E. histolytica* genome (searching E.C. #1.14.13.70). Overall, therefore, it seems that the activity against *E. histolytica* is not mediated through inhibition of an FT enzyme, but could involve another cellular target that is sensitive to this chemotype. Alternatively, FT could be the target in *E. histolytica* as a result of differences in binding preferences compared to other FT enzymes (found in mammals and trypanosomes), but this will need further exploration.

Apart from targeting HMGR in the mevalonate pathway (which is absent in *E. histolytica*), statins exhibit other pleiotropic effects including the modulation of protein prenylation that then leads to the inhibition of the activity of cell signaling molecules including the Rab family of small GTP-binding proteins (Greenwood et al., 2006; Wang et al., 2008). The *E. histolytica* genome encodes heterotrimeric G protein subunits and a large number of small G proteins, which are involved in vesicular trafficking (Bosch and Siderovski, 2013). Accordingly, we measured the effect of statins against *E. histolytica*. Of the statins, mevastatin, atorvastatin, fluvastatin, pitavastatin, and simvastatin tested at 100 μM for 48 h, only simvastatin yielded moderate activity against *E. histolytica*. However, the combination of simvastatin and lonafarnib was slightly synergistic against *E. histolytica*, an intriguing finding given the apparent lack of a HMGR in the parasite.

Using microscopical observation and a constrained scoring system, lonafarnib, tipifarnib and the panel of 125 tipifarnib analogs were also screened in a time and/or concentration-dependent manner against *S. mansoni* somules and adults. Both lonafarnib and tipifarnib were among the most active Group A compounds, causing severe degenerative changes after 24 h in both developmental stages. FT activity has been documented in schistosomes and Ras farnesylation in *S. mansoni* extracts has been inhibited by the FT inhibitor, FTI-277 (Osman et al., 1999). However, like the situation with *E. histolytica*, the tipifarnib analogs that were most active against the schistosome are weak inhibitors of trypanosome and rat FTs (Buckner et al., 2000). This includes about half of the compounds shown in Table 7 (CHN-15, JK-02, JK-17, JK-19, JK-25, JK-35, PN-077, and PN-149; unpublished data). It is possible that the *S. mansoni* FT ortholog is sufficiently distinct from the FTs of other species to limit the above SAR analysis. In this regard, it is worth noting that the three main schistosome species infecting humans all carry a FT gene, but the percentage identities are modest with approximately 36% identity across 79% of the human FT sequence (PF49354.1). In addition, schistosomes do not express CYP51 and, as might be anticipated, there was no correlation between the activity against *S. mansoni* and the EC_50_ values previously measured for activity against *T. cruzi* in which the target is known to be CYP51 (Kraus et al., 2009). Thus, in spite of the potent anti-schistosomal activities measured, understanding the putative target(s) in *S. mansoni* for the tipifarnib analogs will need further investigation.

**Table 7:** First pass screening of FTIs at 10 μM against *S. mansoni* somules and adults.

**Compound**	**Somule severity score at 48 h**	**Adult severity score at 48 h**
Lonafarnib*	4	4
Tipifarnib*	4	4
CHN-13*	4	4
CHN-15	4	4
HB-19	4	4
HB-20	4	4
HB-23	4	4
HB-26	4	4
JK-02*	4	1
JK-17*	4	2
JK-19*	4	4
JK-24	4	4
JK-25	4	4
JK-31	4	4
JK-35	4	4
PN-149	4	3
PN-077	4	4

*Screens were performed twice each in duplicate and representative data are shown. Compounds with asterisks were also those most potent against E. histolytica in first pass screens (Table 1)*.

For both parasites, an analysis of the functional groups on the tipifarnib scaffold showed that large, hydrophobic groups such as 3-phenyl, 3-methyl-phenyl, 4-dimethyl, and naphthalene in the ring 2 position were more active. An analysis of the hydrophobicity of the 125 compounds (cLogP) confirmed a trend that more hydrophobic compounds were associated with stronger anti-amebic and anti-schistosomal activity (Tables 5, 6). It is possible that the higher cLogP values (associated with greater lipophilicity) better facilitated permeability of *E. histolytica* and *S. mansoni*. Previously, for the latter parasite, the bioactivity of six statin analogs was positively associated with lipophilicity (Rojo-Arreola et al., 2014).

Although FTIs showed moderate activity against *E. histolytica* with EC_50_ values ranging from 14.2 to 27.6 μM, we see potential in the use of FTIs in combination with metronidazole. The combination of lonafarnib and metronidazole at 1:1, 1:2, 1:4, 1:8, 2:1, 4:1, 8:1, 16:1 elicited synergism with CI values ranging from 0.5 to 0.8. Thus, the dose of either one or both compounds necessary for complete growth inhibition of the parasite can be reduced.

To conclude, we report the potent anti-amebic and anti-schistosomal activities of the clinically developed FTIs, lonafarnib and tipifarnib. Screening of a previously developed tipifarnib analog library comprising 125 compounds identified subsets of potent compounds with little overlap between *E. histolytica* and *S. mansoni*. Also, scrutiny of the SAR data available for this set of compounds against trypanosomatid parasites, suggests that neither the *E. histolytica* nor the *S. mansoni* FT seems to be the target responsible for the bioactivities recorded. Nonetheless, the potent bioactivities measured encourage further study. Finally, the synergistic activity of the combination of lonafarnib and metronidazole against *E. histolytica* provides an opportunity to investigate a combination therapy for amebiasis.

## Author Contributions

AP performed experiments, analyzed the data, and prepared the original draft. TN performed experiments and analyzed the data. NE-S, DS, and BS provided resources. FB analyzed the data, reviewed and edited the manuscript. MG synthesized the farnesyltransferase inhibitors. CC conceptualized the study, performed experiments, analyzed the data, reviewed and edited the manuscript. AD conceptualized the study, performed experiments, analyzed the data, wrote, reviewed and edited the manuscript.

### Conflict of Interest Statement

The authors declare that the research was conducted in the absence of any commercial or financial relationships that could be construed as a potential conflict of interest.

## Supplementary Material

Supplementary Table 1 Chemical structures and properties of analogs of tipifarnib and their activity against *E. histolytica* trophozoites. NA means compound was not available for testing.

Supplementary Table 2 Chemical structures of tipifarnib analogs and their activity against *S. mansoni* somules and adults.

Supplementary Video 1 DMSO control adult *S. mansoni* incubated for 24 h. Three male parasites are visible. Note the flexibility of the parasites and their ability to grasp the well bottom with their oral and ventral suckers.

Supplementary Video 2 Adult *S. mansoni* incubated for 24 h in the presence of 10 μM tipifarnib. Five male parasites are visible in the movie. Relative to the DMSO control parasites (Supplementary Video 1), note the uncoordinated movement, more constrained flexibility, darkened appearance and the inability of the parasites to adhere to the well bottom with their oral and ventral suckers.
